# Secure and Lightweight Cluster-Based User Authentication Protocol for IoMT Deployment †

**DOI:** 10.3390/s24227119

**Published:** 2024-11-05

**Authors:** Xinzhong Su, Youyun Xu

**Affiliations:** School of Communication and Information Engineering, Nanjing University of Posts and Telecommunications, Nanjing 210003, China; suxinz20@163.com

**Keywords:** Internet of Medical Things, mutual authentication, fine-grained access control, security

## Abstract

Authentication is considered one of the most critical technologies for the next generation of the Internet of Medical Things (IoMT) due to its ability to significantly improve the security of sensors. However, higher frequency cyber-attacks and more intrusion methods significantly increase the security risks of IoMT sensor devices, resulting in more and more patients’ privacy being threatened. Different from traditional IoT devices, sensors are generally considered to be based on low-cost hardware designs with limited storage resources; thus, authentication techniques for IoMT scenarios might not be applicable anymore. In this paper, we propose an efficient three-factor cluster-based user authentication protocol (3ECAP). Specifically, we establish the security association between the user and the sensor cluster through fine-grained access control based on Merkle, which perfectly achieves the segmentation of permission. We then demonstrate that 3ECAP can address the privilege escalation attack caused by permission segmentation. Moreover, we further analyze the security performance and communication cost using formal and non-formal security analysis, Proverif, and NS3. Simulation results demonstrated the robustness of 3ECAP against various cyber-attacks and its applicability in an IoMT environment with limited storage resources.

## 1. Introduction

The number of connected devices has grown exponentially due to advances in communications technology, resulting in what is known as the Internet of Things (IoT) [1,2,3]. IOT technology has continued to develop and innovate, profoundly changing traditional industrial models and people’s lifestyles, such as smart agriculture, smart healthcare, smart homes, and self-driving cars [4]. And healthcare is rapidly evolving, driven by an aging population, consumer demand for better services in more affordable prices, and a growing global focus on preventative health [5,6]. In recent years, IoMT has been recognized as one of the most important technologies in healthcare, which is used for systematic monitoring of patient status, enabling doctors to provide timely and appropriate treatment [7]. Specifically, IoMT sensors such as defibrillators, sphygmomanometers, and oximeters provide real-time monitoring and observation for patients’ temperature, pulse, blood pressure, respiration, and more [8]. Typically, sensors in IoMT are widely accessible and can be installed across geographies as the focus has been on making them multifunctional, low-cost, and available on hardware platforms when coordinated with back-end processing systems. With these new technologies, the prospect of IoMT sensors in healthcare is extremely promising.

Despite the convenience that IoMT brings to patients in terms of treating, diagnosing, and maintaining their health, once the information carried by these sensors is accessed by attackers, it can be a great threat to patient privacy and security [9]. One of the crucial factors to ensure the security of IoMT is node authentication. Usually, the generic architecture of IoMT consists of three node participants, i.e., user, gateway, and sensor. The sensor is placed in a designated area to collect environmental parameters and then transmits these parameters to the gateway through a wireless channel [10]. The user must be authenticated to access these data as the patient data provided by sensors are analyzed and collated to make appropriate and feasible decisions for the timely treatment of the patient.

Specifically, the IoMT system can be simplified into three dimensions, i.e., perception layer, network layer, and application layer [11]. (1) Perception layer: each patient is equipped with a variety of medical sensors used to sense and monitor vital statistics. In this layer, the attacker usually utilizes a device capture attack to obtain patient information inside sensors. (2) Network layer: similar to the OSI network and transport layer, it is responsible for authentication, communication and data transfer between sensors and users via an open channel/private network. However, it is vulnerable to man-in-the-middle attacks, impersonation attacks, replay attacks and so on. (3) Application layer: in this layer, legitimate users/medical staff can realize access to patient information through authentication with the sensors, and it is the top layer of the three-layer IoMT system architecture. However, the application layer is also vulnerable to many attacks such as insider privilege attacks, privilege escalation attacks, etc. Therefore, it is necessary to ensure the privacy and security of patient information in the multilayer architecture of an IoMT system.

Once the information is compromised, the corresponding patient information (including history of illness) may also be exposed to the attacker [12]. Worse, the attacker can even illegally sell this information, thus seriously compromising the patient’s personal privacy. In addition, insider attackers (i.e., medical staff) also pose a potential risk of IoMT information leakage. It is extremely necessary to implement permission segmentation according to access levels due to the differences in sensor data accessible to medical staff in different departments (e.g., neurology, gastroenterology, cardiovascular, etc.). Moreover, IoMT is susceptible to various types of attacks, including replay attacks, user privilege escalation attacks, smart card theft attacks, etc., which further compromise the security of the system. Therefore, it is urgent to design a new authentication protocol to ensure the security and privacy of IoMT.

Considering the security, low complexity, and low cost requirements of IoMT, we propose a new efficient cluster-based lightweight secure authentication protocol (3ECAP), with the ultimate goal of establishing a secure session key before participants transmit data. The specific contributions of this paper are as follows.

(1) 3ECAP implements IoMT user permission segmentation using fine-grained access control to establish a security association between the user and the sensor cluster, which reduces subsequent database access costs. Then, the user’s password, biometrics and smart card are used as the three factors for authentication, where biometrics are collected through a fuzzy extractor. In addition, the communication cost and computation cost of 3ECAP are further reduced by only performing hash and dissimilarity operations.

(2) The formal security analysis of 3ECAP is demonstrated through the widely used Real or Random (RoR) model and the formal automated verification tool Proverif. In addition, 3ECAP informal security analysis is also provided, which indicates that 3ECAP is not only resistant to most known attacks but also to privilege elevation attacks from insiders (see Section 6.2).

(3) Considering the limited resources of IoMT devices, compared to other schemes, our proposed authentication protocol is not only lightweight and efficient but also resistant to a variety of complex typical attacks.

The rest of this paper is structured as follows: Section 2 presents the literature survey. Some necessary mathematical background is provided in Section 3. The system model utilized in 3ECAP is given in Section 4. Section 5 describes the phases of the designed protocol (3ECAP). In Section 6, the security of 3ECAP is ensured by using formal and informal security analysis. Section 7 presents a comparative analysis of 3ECAP and other related protocols with respect to computational cost, latency, and security characteristics. Section 8 presents a simulation analysis of 3ECAP using a network simulation tool. The last section concludes the paper and gives some future research directions.

## 2. Related Work

In this section, research advances in the relevant areas are provided, including the methods used and advantages and limitations.

Wang et al. [13] proposed a cloud-assisted secure user authentication scheme with various attributes such as forward secrecy and multi-factor security. However, the scheme requires high computational costs and does not ensure user privacy. Masud et al. [14] proposed a lightweight anonymous user authentication protocol for IoT, which only uses lightweight cryptographic primitives (hash). The scheme establishes a secure session for legitimate users and prohibits unauthorized user access to IoT sensor nodes. Although the protocol has low computational and communication costs, it proved to be vulnerable to attacks such as impersonation and replay. In addition, relevant existing protocols [15,16,17] are designed for various IoT scenarios, e.g., IoMT, smart firefighting, smart transportation, etc., with provably secure protocols that provide mutual authentication for involved nodes. However, according to recent studies [18,19,20], the mentioned schemes are susceptible to attacks such as man-in-the-middle, denial-of-service, and internal privilege.

Zhang et al. [21] propose a password-based lightweight security authentication scheme that can flexibly achieve mutual authentication between the user and sensor. Unfortunately, studies have demonstrated that this authentication scheme based on only a single factor can be easily compromised and therefore cannot withstand attacks such as password guessing. To address these problems, Nandy et al. [22] and Singh et al. [23] have proposed security schemes based on multifactor privacy protection. However, Chaudhry et al. [24] point out that the public key of the sensor in the scheme of Nandy et al. [22] is invalid, due to the inability of the device to generate its own private key, and susceptible to clogging attacks. Moreover, the above schemes also require high communication costs.

Nyangaresi et al. [25] propose a lightweight key management and mutual authentication protocol based on Elliptic Curve Cryptography (ECC) for smart home environments. Li et al. [26] design a robust two-factor user authentication protocol based on ECC and prove that the construction of the proposed scheme can achieve user anonymity, forward secrecy of the session key, etc. However, since the above schemes use the ECC algorithm, this significantly increases the communication and computational costs to verify the protocol. Furthermore, Xie et al. [27] proposed a blockchain-based vehicle-to-infrastructure (V2I) authentication protocol using lightweight cryptographic primitives that guarantee sensor anonymity and untraceability. Son et al. [28] design a lightweight mutual authentication protocol for IoT sensors, in which the node performs cryptographic computation only when switching in order to improve the network transmission efficiency. Yang et al. [29] propose a mutual authentication scheme based on decentralized edge collaboration to provide continuous protection for zero-trust IoT and enable flexible updating for the sensor.

By reading and summarizing the above existing studies, we found that existing authentication protocols have low utility in IoMT, e.g., susceptibility to various attacks, high overhead algorithmic application, access control of user authority, high maintenance cost of protocol, and so on. Therefore, we intended to design a lightweight secure and reliable authentication protocol for IoMT to solve the above problems, and some of these research results have been published in the form of a conference [30]. Please note that 3ECAP is an extended version of the published conference paper. Compared to the previous version, 3ECAP contains more comprehensive authentication schemes, security analyses, simulations, graphs, results and utilities. The relevant changes are indicated in the text. Table 1 summarizes the relevant work described above.

## 3. Preliminaries

### 3.1. One-Way Hash Function

A one-way hash function can transform an input message string of arbitrary length into a fixed-length output. It is widely used in areas such as the generation of message digests and message authentication codes, key encryption, and data integrity tests. Collision resistance is the main property and is defined as follows.

**Definition** **1.** Suppose a one-way hash function can be expressed as $h:{\left\{0,1\right\}}^{*}\to {\left\{0,1\right\}}^{n}$. Specifically, the hash function outputs a fixed-length binary string $h\left(m\right)\in {\left\{0,1\right\}}^{n}$ for an arbitrary-length input binary string $m\in {\left\{0,1\right\}}^{*}$. Assume $Adv_{A}^{HASH}\left(t\right)$ is defined as the probability of an adversary obtaining a hash collision in execution time t, then $Adv_{A}^{HASH}\left(t\right)=\mathrm{Pr}\left[\left(m,n\right)\in_{R}A:m\neq n,h\left(m\right)=h\left(n\right)\right]$, where $P_{r}\left[X\right]$ refers to the probability of a random event X occurring, and $\left(m,n\right)\in_{R}A$ means that both input strings m and n are randomly selected by A. If an (θ,t)-adversary A attempts to attack the collision resistance of h(·), it means that the maximum execution time of A is t and that $\mathrm{Ad}v_{\left(A\right)}^{\mathrm{HASH}}\left(t\right)\leq \theta$.

### 3.2. Fuzzy Extractor for Biometric Verification

The secret value in an encryption mechanism is a random string that requires uniform distribution and can be copied exactly. However, in the real world, it is difficult for the secret value to satisfy this. For example, biometric features, such as fingerprints, brain prints, etc., cannot be accurately copied due to a non-uniform distribution of random values. Thus, we select the fuzzy extraction method for the collection of biometric features [31].

Recently, the fuzzy extractor method has been widely used to extract biometric keys from user biometric input. This method can allow the input to have a certain amount of noise (or error), and as long as the input is similar, the same uniform random string can be extracted. The general structure is as follows.

(1) Gen: Given that the user inputs biometrics $BIO_{i}$, the gen process will generate a biometric key $r_{i}$ of *l* bits and the corresponding auxiliary public parameter $p_{i}$; that is, $Gen\left(BIO_{i}\right)=\left(r_{i},p_{i}\right)$.

(2) Rep: Given a noisy user input biometric $BIO_{i}^{\prime }$, Rep will return the original biometric key $r_{i}$ with the help of the auxiliary public data $p_{i}$ when the Hamming distance between the current biometric input $BIO_{i}^{\prime }$ and the original biometric input $BIO_{i}$ is less than a specific error tolerance threshold *t*; that is, $HamDis(BIO_{i}^{\prime },BIO_{i})\leq t$. Thus, $Rep\left(BIO_{i}^{\prime },p_{i}\right)=\left(r_{i}\right)$.

Considering the false-positive and false-negative events of biometric authentication, we make a note of $BIO_{i}$ and $BIO_{i}^{\prime }$. If both $BIO_{i}$ and $BIO_{i}^{\prime }$ originate from the same person, then the Hamming distance between the two will converge to 0. We assume that $Pr[HamDis\left(BIO_{i}^{\prime },BIO_{i}\right)\leq t]\geq 1-\lambda n$, where λn means the false negative probability. If $BIO_{i}$ and $BIO_{i}^{\prime }$ originate from different people, then the Hamming distance between the two may be significant. We assume that $Pr[HamDis\left(BIO_{1},BIO_{2}\right)\geq t^{\prime }]\geq 1-\lambda p$, $t^{\prime }\gg t$, where λp means the false positive probability.

## 4. System Model

### 4.1. Authentication Model

**Figure 1:**
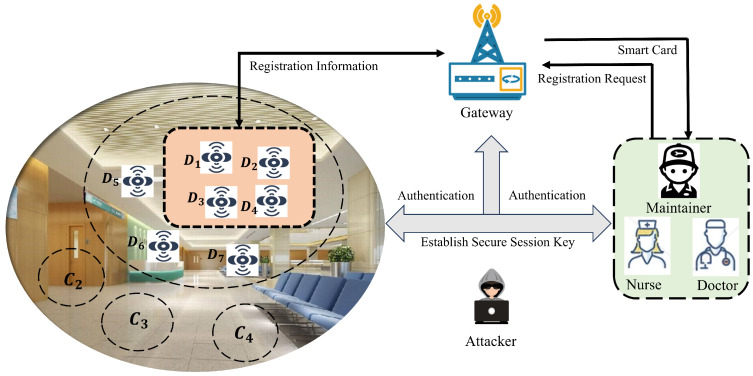
Authentication model for IoMT.

The IoMT-based authentication model is shown in Figure 1. In this model, patients suffering from different diseases are being treated in the hospital. Each hospital bed is equipped with a number of sensors to monitor and sense the real-time status of the patient (e.g., blood pressure, heart rate, etc.). Since the hospital contains different departments, such as brain, orthopedics, etc., as well as different types of medical staff in each department, such as doctors and nurses, they are all concerned with monitoring the patient’s physical condition. Specifically, only the nurse is required to handle a patient who needs a medication change, while the doctor is required to take quick emergency measures when the patient is in a life-threatening situation. Therefore, it is necessary to set the corresponding accessible sensor cluster for different user levels.

Four different departments $C_{1}$, $C_{2}$, $C_{3}$, and $C_{4}$ exist in the hospital, as shown on the left side of Figure 1, and some sensor devices are deployed in them. For example, in $C_{1}$, seven sensors $\left\{D_{1},D_{2},...,D_{7}\right\}$ are deployed to detect real-time data of patients, where $\left\{D_{1},D_{2},D_{3},D_{4}\right\}$ represents the accessible sensor cluster by a particular member of the medical staff $U_{1}$. Before authentication, both $U_{1}$ and $D_{j}$ need to complete registration with the help of GW, where $U_{1}$ also sends a sensor cluster to GW. Then, $U_{1}$ can authenticate with $D_{j}$ through GW. Once authenticated, $U_{1}$ can securely access the real-time data from $D_{j}$. Specifically, $U_{1}$ first sends a login request to GW. Then, GW validates the login request and sends the access request to the accessible $D_{j}$. Finally, once the authentication is complete, $D_{j}$ sends a reply message to $U_{1}$ and generates a session key shared between the two. It is worth noting that the registration phase of 3ECAP is performed in a secure environment, whereas information is transmitted via a public channel in the authentication phase, which makes it vulnerable to anonymous attackers.

### 4.2. Threat Model

The protocol we designed uses the Dolev–Yao [32] threat model (DY model) for security analysis, where an adversary can not only intercept messages transmitted between participants but also perform deletion and modification operations. In addition, we consider the widely accepted RoR model [33], which is used to secure the session key generated by medical staff and sensors. Note that in the authentication model, suppose that the GW is fully trusted and is deployed in a fixed location that is physically protected so that the likelihood of the GW being captured is extremely low compared to that of the sensor device. In contrast, for some physically captured sensor devices, the corresponding secret information stored in these devices can be extracted by the adversary using power analysis attacks.

## 5. Proposed Scheme

In this section, we elaborate on a new protocol called 3ECAP for IoMT deployments. The protocol requires the following phases: (1) setup; (2) medical staff registration; (3) sensor registration; (4) login and authentication; (5) password and biometric update; and (6) new smart-device addition phase. In the setup phase, the public parameters of the protocol are selected by the fully trusted GW. Once the setup is complete, the medical staff and the sensor need to complete the registration in the system. In the login and authentication phase, a user (i.e., legal medical staff) $U_{i}$ and a sensor device $SD_{j}$, with the help of the GW, establish a shared key between $U_{i}$ and $SD_{j}$ for future communication. The proposed protocol also enables $U_{i}$ to change the password and biometric information without the need for GW. In addition, the protocol can support the addition of new sensor devices. The notations and their abbreviations are presented in Table 2 [30] for the analysis of 3ECAP.

### 5.1. Setup Phase

During the system setup phase, some public parameters are initialized by GW. Specifically, GW chooses a one-way hash function h(·), a biometric key generation function Gen(·) and a biometric key replication function Rep(·), where Gen(·) and Rep(·) are used for bio-information extraction and recovery of medical staff, respectively. Then, GW generates a unique master key *x*, an identity $ID_{GW}$, and also calculates the corresponding pseudo-identity $RID_{GW}=h\left(ID_{GW}∥x\right)$.

### 5.2. Sensor Addition Phase

During the sensor addition phase, GW generates a unique $SID_{j}$ for the medical sensor $SD_{j}$, a random number $\alpha_{j}$, and then calculates the pseudo-identity $RSID_{j}=h(SID_{j}∥ID_{GW}∥\alpha_{j})$. In addition, a secret pairwise key is established between GW and $SD_{j}$ by means of the master key *x* of GW, where $k_{GWj}=h(ID_{GW}∥SID_{j}∥x)$, which will be used for mutual authentication and message encryption between nodes in the subsequent login phase. Finally, GW stores $\left\{RSID_{j},k_{GWj}\right\}$ into the database. Meanwhile, $SID_{j}$ also saves $\left\{RSID_{j},k_{GWj}\right\}$ into the memory.

### 5.3. Medical Staff Registration Phase

In general, there are many disease departments in the medical system, such as neurology, orthopedics, brain and cardiovascular, etc. Each department is composed of many medical sensor devices that contain sensitive patient information. Therefore, in order to protect patient privacy, medical staff can only access patient information based on access permission for a specific cluster of sensor devices. The registration process for medical staff can also be divided into two phases, as follows.

(1) Fine-Grained Access Control: The purpose of fine-grained access control [30] is to restrict the access permission of the medical staff. For example, medical staff in a neurology department can only access information from sensors relevant to their department, where these sensors are connected to the patient to monitor individual status.

Our sensor cluster model can be simplified to a merkle tree, which consists of multiple leaf nodes $\left\{D_{1},D_{2},...,D_{n}\right\}$ and a single root node $Ver_{i}$, where $\left\{D_{1},D_{2},...,D_{n}\right\}$ represents the sensor device nodes accessible to a particular medical staff. Assume that the number of leaf nodes $n=2^{m}$ for m≥1, $Ver_{i}$ can be computed as follows.

Procedure 1: Denote the leaf nodes $D_{1},D_{2},...,D_{n}$ as $H_{\left(log_{2}n\right)\left(0\right)},H_{\left(log_{2}n\right)\left(1\right)},...,H_{\left(log_{2}n\right)\left(n-1\right)}$, respectively.

Procedure 2: $Ver_{i}=H_{00}=h\left(H_{10}∥H_{11}\right)$, where $H_{xy}=h\left(H_{\left(x+1)(2y\right)}∥H_{\left(x+1)(2y+1\right)}\right)$ for $x=0,1,2,...,(\log_{2}n)-1$ and y=0,1,2,...,n−1.

**Figure 2:**
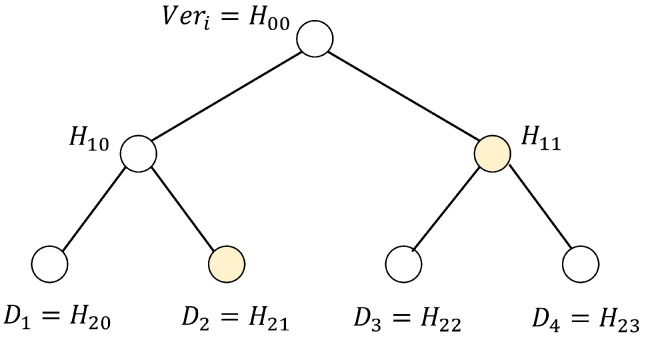
Merkle tree-based access list.

$Ver_{i}$ can also be calculated using auxiliary and leaf nodes, which can effectively reduce the computational complexity. As shown in Figure 2 [30], $Ver_{i}=h\left(h\left(H_{21}∥H_{20}\right)∥H_{11}\right)$, utilizing auxiliary nodes $H_{20}$ and $H_{11}$, instead of $H_{22}$ and $H_{23}$.

(2) *Personal Information Registration*

Step 1: $U_{i}$ chooses his or her identity $ID_{i}$, password $PW_{i}$, access list (generated from the cluster of accessible sensors) $AL=\left\{RSID_{1},RSID_{2},...,RSID_{j}\right\}$ and imprints biological information $BIO_{i}$ on the specific acquisition device. The device then extracts the secret parameter $r_{i}$ and the public parameter $p_{i}$ with the help of the generating function Gen(·), namely $Gen\left(BIO_{i}\right)=\left(r_{i},p_{i}\right)$. Next, $U_{i}$ computes $RPW_{i}=h\left(PW_{i}∥r_{i}\right)$ and sends $\left\{ID_{i},RPW_{i},AL\right\}$ to GW via a secure channel.

Step 2: After receiving the message $\left\{ID_{i},PW_{i},AL\right\}$, GW computes $Ver_{i}$ using the above markle tree and auxiliary nodes and also computes the personal information $P_{i}=h\left(ID_{i}∥RPW_{i}\right)$. Moreover, GW generates the current registration timestamp $T_{re}$, a random number $\beta_{i}$, and computes the pseudo-identity $RID_{i}=h(ID_{i}∥ID_{GW}∥\beta_{i})$ and the sensor device list $SDL_{i}=h(Ver_{i}∥T_{re}∥RID_{i})$. Next, GW stores $\left\{RID_{i},SDL_{i}\right\}$ in its memory. GW also returns the message $\left\{P_{i},RID_{i},SDL_{i},RID_{GW}\right\}$ to $U_{i}$ via a secure channel.

**Figure 3:**
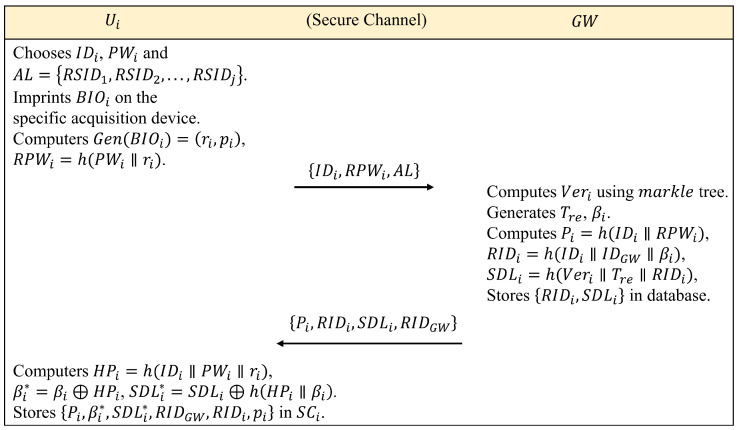
Summary of medical staff registration phase.

Step 3: Once the message is received from GW, $U_{i}$ calculates $HP_{i}=h(ID_{i}∥PW_{i}∥r_{i})$, $\beta_{i}^{*}=\beta_{i}⊕HP_{i}$, $SDL_{i}^{*}=SDL_{i}⊕h\left(HP_{i}∥\beta_{i}\right)$. Finally, $U_{i}$ stores the verifiable information $\left\{P_{i},\beta_{i}^{*},SDL_{i}^{*}RID_{GW},RID_{i},p_{i}\right\}$ into its own smart card $SC_{i}$; note that $SDL_{i}^{*}$ represents the cluster of sensor devices accessible to the particular user. Figure 3 illustrates the complete process of 3ECAP registration.

### 5.4. Login and Authentication Phase

When $U_{i}$ wants to access the data of $SD_{j}$, he/she needs to login and authenticate to the GW first. After the authentication process is complete, a secure session key is established between $U_{i}$ and $SD_{j}$ for subsequent communication. The following steps are essential under the proposed protocol.

Step 1: $U_{i}\to GW:\left\{A_{i},B_{i},C_{i},RSID_{j},T_{1}\right\}$

Step 1.1: $U_{i}$ inputs $ID_{i}^{\prime }$, $PW_{i}^{\prime }$, and imprints $BIO_{i}^{\prime }$ at a biometric acquisition device. $SC_{i}$ then extracts the public parameter $p_{i}$ and recovers $r_{i}=Rep\left(BIO_{i}^{\prime },p_{i}\right)$ if $HamDis(BIO_{i}^{\prime },BIO_{i})\leq t$ is satisfied. Next, $SC_{i}$ computes $P_{i}^{\prime }=h\left(ID_{i}^{\prime }∥h\left(PW_{i}^{\prime }∥r_{i}\right)\right)$ and verifies $P_{i}^{\prime }\overset{?}{=}P_{i}$. The login request is terminated if $P_{i}^{\prime }\neq P_{i}$.

Step 1.2: $SC_{i}$ then generates the current timestamp $T_{1}$, a random number *a*, and calculates $HP_{i}=h(ID_{i}^{\prime }∥PW_{i}^{\prime }∥r_{i})$, $\beta_{i}^{\prime }=\beta_{i}^{*}⊕HP_{i}$, $SDL_{i}^{\prime }=SDL_{i}^{*}⊕h\left(HP_{i}∥\beta_{i}^{\prime }\right)$, $A_{i}=RID_{i}⊕h\left(RID_{GW}∥T_{1}\right)$, $b_{i}=h(RID_{i}∥T_{1}∥a)$, $B_{i}=h(RID_{i}∥RID_{GW}∥T_{1})⊕b_{i}$, $C_{i}=h(b_{i}∥SDL_{i}^{\prime }∥RID_{GW}∥RSID_{j}∥T_{1})$. Finally, $SC_{i}$ sends the message $M_{1}=\left\{A_{i},B_{i},C_{i},RSID_{j},T_{1}\right\}$ to GW via a common channel, where $RSID_{j}$ contains the information $U_{i}$ wants to obtain.

Step 2: $GW\to SD_{j}:\left\{D_{i},E_{i},F_{i},T_{2}\right\}$

Step 2.1: Once $M_{1}$ is received from $U_{i}$, GW first verifies the validity of $T_{1}$ under the condition of $\left|T_{1}^{*}-T_{1}\right|\leq \Delta T$, where $T_{1}^{*}$ is the receive timestamp, and ΔT is the maximum time delay. The entire session is aborted if the condition is not met. Otherwise, GW computes $RID_{i}=A_{i}⊕h\left(RID_{GW}∥T_{1}\right)$ and finds the corresponding $SDL_{i}$ from the memory. Meanwhile, GW also calculates $b_{i}=B_{i}⊕h(RID_{i}∥RID_{GW}∥T_{1})$, $C_{i}^{\prime }=h(b_{i}∥SDL_{i}∥RID_{GW}∥RSID_{j}∥T_{1})$ and verifies $C_{i}^{\prime }\overset{?}{=}C_{i}$. If $C_{i}^{\prime }\neq C_{i}$, it indicates two possibilities, *case 1:* $U_{i}$ is an external attacker who does not have the key for the registration phase of the personnel information, and *case 2:* $U_{i}$ is an internal attacker who wants to access sensors beyond his/her own permission, i.e., the sensor cluster $SDL_{i}^{\prime }\neq SDL_{i}$.

Step 2.2: If $C_{i}^{\prime }=C_{i}$, it indicates that the identity of $U_{i}$ is confirmed. Then, GW generates the current timestamp $T_{2}$, a random number *b* and computes $D_{i}=b⊕h\left(k_{GWj}∥T_{2}\right)$, $E_{i}=b_{i}⊕h\left(b∥T_{2}\right)$, $F_{i}=h(RSID_{j}∥k_{GWj}∥b_{i}∥b∥T_{2})$, where $k_{GWj}$ is the symmetric key for $SD_{j}$ and is stored in the memory of GW. At last, GW sends the message $M_{2}=\left\{D_{i},E_{i},F_{i},T_{2}\right\}$ publicly to $SD_{j}$.

Step 3: $SD_{j}\to U_{i}:\left\{G_{i},H_{i},J_{i},T_{3}\right\}$

Step 3.1: Once $SD_{j}$ receives message $M_{2}$ from GW, $SD_{j}$ verifies that timestamp $T_{2}$ matches condition $\left|T_{2}^{*}-T_{2}\right|\leq \Delta T$. If the condition does not match, it indicates that the timeliness of $M_{2}$ is not guaranteed and the session will be closed. Otherwise, $SD_{j}$ calculates $b=D_{i}⊕h\left(k_{GWj}∥T_{2}\right)$, $b_{i}=E_{i}⊕h\left(b∥T_{2}\right)$ and $F_{i}^{\prime }=h(RSID_{j}∥k_{GWj}∥b_{i}∥b∥T_{2})$ using the stored symmetric key $k_{GWj}$. $SD_{j}$ then verifies that $F_{i}^{\prime }\overset{?}{=}F_{i}$.

Step 3.2: If $F_{i}^{\prime }\neq F_{i}$, the access request from GW is terminated. Otherwise, $SD_{j}$ authenticates GW successfully. Then, $SD_{j}$ generates the current timestamp $T_{3}$, a random number *c* and calculates $G_{i}=c⊕b_{i}$, $H_{i}=c⊕h\left(k_{GWj}∥T_{2}\right)=c⊕d_{i}$, $J_{i}=h(RSID_{j}∥d_{i}∥c∥T_{3})$. Meanwhile, $SD_{j}$ also computes the secure session key $sk_{ji}=h(b_{i}∥d_{i}∥RSID_{j}∥c∥T_{3})$. Finally, $SD_{j}$ sends the message $M_{3}=\left\{G_{i},H_{i},J_{i},T_{3}\right\}$ to $U_{i}$ via a public channel.

**Figure 4:**
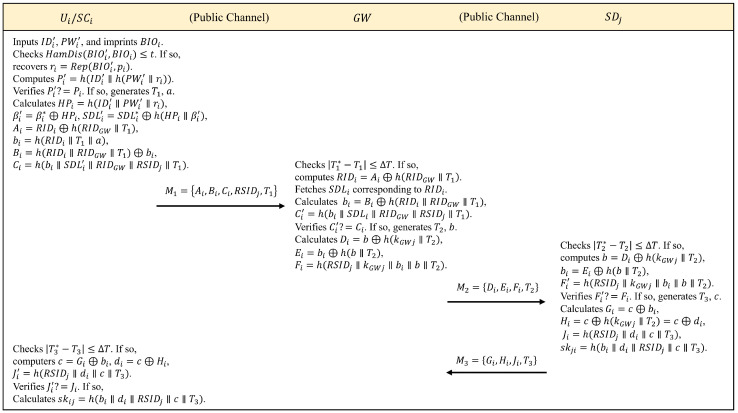
Summary of login and authentication phase.

Step 4: Once $M_{3}$ is received at time $T_{3}^{*}$ by $U_{i}$, $SC_{i}$ verifies the validity of $T_{3}$ in this message with the condition of $\left|T_{3}^{*}-T_{3}\right|\leq \Delta T$. If the condition fails, the session is immediately terminated by $U_{i}$. Otherwise, $SC_{i}$ calculates $c=G_{i}⊕b_{i}$, $d_{i}=c⊕H_{i}$ and $J_{i}^{\prime }=h(RSID_{j}∥d_{i}∥c∥T_{3})$ and verifies $J_{i}^{\prime }\overset{?}{=}J_{i}$. If $T_{i}^{\prime }=J_{i}$, it means that the identity of $SD_{j}$ is confirmed. Eventually, $SC_{i}$ computes the session key $sk_{ij}=h(b_{i}∥d_{i}∥RSID_{j}∥c∥T_{3})\left(=sk_{ji}\right)$ shared with $SD_{j}$, which will be used to encrypt the data transmitted between $U_{i}$ and $SD_{j}$. Figure 4 illustrates the complete process of 3ECAP login and authentication.

### 5.5. Password and Bio-Information Update Phase

Usually, human biological characteristics change over time, for example, the characteristics of brain waves are completely different at different ages. Therefore, 3ECAP supports the modification of biological information for medical staff. In addition, we recommend that medical staff change their passwords regularly to ensure the security of their privacy (this part was not considered in the previous conference). The specific steps for password and bio-information modification are as follows.

Step 1: $U_{i}$ input $ID_{i}$ and $PW_{i}^{old}$ and imprint the old bio-information $BIO_{i}^{old}$ on the specific collection device. Meanwhile, $U_{i}$ inserts its smart card $SC_{i}$ in the system terminal. Then, $SC_{i}$ computes $r_{i}^{old}=Rep\left(BIO_{i}^{old},p_{i}\right)$ with the condition $HamDis(BIO_{i}^{old},BIO_{i})\leq t$, where $BIO_{i}$ is the biological information previously registered by $U_{i}$. Next, $SC_{i}$ computes $P_{i}^{old}=h\left(ID_{i}∥h\left(PW_{i}^{old}∥r_{i}^{old}\right)\right)$ and verifies $P_{i}^{old}\overset{?}{=}P_{i}$. If $P_{i}^{old}=P_{i}$, $SC_{i}$ authenticates $U_{i}$ successfully. Otherwise, password and bio-information change requests are terminated by $SC_{i}$.

Step 2: After successful authentication, $U_{i}$ enters a new password $PW_{i}^{new}$ and imprints the new bio-information $BIO_{i}^{new}$ at the acquisition device. The device then extracts the corresponding secret parameter $r_{i}^{new}$ and public parameter $p_{i}^{new}$ using Gen(·). Next, $SC_{i}$ calculates the old secret information $HP_{i}^{old}=h(ID_{i}∥PW_{i}^{old}∥r_{i}^{old})$, $\beta_{i}=\beta_{i}^{*}⊕HP_{i}^{old}$ and $SDL_{i}=SDL_{i}^{*}⊕h\left(HP_{i}^{old}∥\beta_{i}\right)$. $SC_{i}$ also calculates the new secret information $P_{i}^{new}=h\left(ID_{i}∥h\left(PW_{i}^{new}∥r_{i}^{new}\right)\right)$, $HP_{i}^{new}=h(ID_{i}∥PW_{i}^{new}∥r_{i}^{new})$, $\beta_{i}^{new}*=\beta_{i}⊕HP_{i}^{new}$, $SDL_{i}^{new}*=SDL_{i}⊕h\left(HP_{i}^{new}∥\beta_{i}\right)$. $SC_{i}$ finally replaces $\left\{P_{i},\beta_{i}^{*},SDL_{i}^{*},p_{i}\right\}$ with $\left\{P_{i}^{new},\beta_{i}^{new}*,SDL_{i}^{new}*,p_{i}^{new}\right\}$ in its memory.

### 5.6. New Smart Device Addition Phase

Usually, a sensor is installed in each sickbed to capture the real-time status of the patient (e.g., blood pressure, temperature, heartbeat, etc.). Hence, the number of sensors is generally fixed. However, when emergencies arise (for example, the outbreak of COVID-19), the original number of beds cannot meet the demand of patients. Therefore, 3ECAP can support the bulk addition of new sensors with the following steps (this part was not considered in the previous conference).

Step 1: Before new sensors are deployed, they need to register with the gateway. Specifically, GW selects an identity $SID_{j}^{new}$ and a random number $\alpha_{j}^{new}$ for $SD_{j}^{new}$ and computes the pseudo-identity $RSID_{j}^{new}=h(SID_{j}^{new}∥ID_{GW}∥\alpha_{j}^{new})$. Meanwhile, GW computes $k_{GWj}^{new}=h(ID_{GW}∥SID_{j}^{new}∥x)$. Then, GW and $SD_{j}^{new}$ store $\left\{SID_{j}^{new},RSID_{j}^{new},k_{GWj}^{new}\right\}$ and $\left\{RSID_{j}^{new},k_{GWj}^{new}\right\}$ into their own databases, respectively. Furthermore, after the registration of the sensor is complete, GW needs to broadcast the addition regarding $SD_{j}^{new}$ so that $U_{i}$ can access the data therein.

Step 2: $U_{i}$ needs to update sensor device list $SDL_{i}$ with the help of GW before accessing the bulk-added $\left\{RSID_{1}^{new},RSID_{2}^{new},...,RSID_{j}^{new}\right\}$. $U_{i}$ first inputs $ID_{i}^{\prime }$, $PW_{i}^{\prime }$, $BIO_{i}^{\prime }$ and inserts $SC_{i}$. Then, $SC_{i}$ calculates $r_{i}=Rep\left(BIO_{i}^{\prime },p_{i}\right)$ if condition $HamDis(BIO_{i}^{\prime },BIO_{i})\leq t$ is satisfied. Next, $SC_{i}$ computes $P_{i}^{\prime }=h\left(ID_{i}^{\prime }∥h\left(PW_{i}^{\prime }∥r_{i}\right)\right)$ and verifies $P_{i}^{\prime }\overset{?}{=}P_{i}$. If $P_{i}^{\prime }=P_{i}$, $SC_{i}$ sends $\left\{RID_{i},AL\right\}$ to GW via a secure channel, where AL consists of the old devices $\left\{RSID_{1},RSID_{2},...,RSID_{j}\right\}$ and the newly added devices $\left\{RSID_{1}^{new},RSID_{2}^{new},...,RSID_{j}^{new}\right\}$.

Step 3: Once the message $\left\{RID_{i},AL\right\}$ is received from $U_{i}$, GW generates a new current registration timestamp $T_{re}^{new}$ and computes $SDL_{i}^{new}=h(Ver_{i}^{new}∥T_{re}^{new}∥RID_{i})$, where $Ver_{i}^{new}$ is calculated based on AL using the merkle tree. Then, GW sends $SDL_{i}^{new}$ to $U_{i}$ via a secure channel.

Step 4: When $SDL_{i}^{new}$ is received from GW, $SC_{i}$ computes $HP_{i}=h(ID_{i}^{\prime }∥PW_{i}^{\prime }∥r_{i})$, $\beta_{i}=\beta_{i}^{*}⊕HP_{i}$, $SDL_{i}^{new}*=SDL_{i}^{new}⊕hHP_{i}∥\beta_{i})$. Finally, $SC_{i}$ replaces $SDL_{i}^{*}$ with $SDL_{i}^{new}*$ in its memory.

## 6. Security Analysis

In this section, we verify the security reliability of 3ECAP using both formal and informal security analysis. Specifically, we first prove the security of session keys in the proposed protocol based on the ROR model. Then, we use informal security analysis to demonstrate that 3ECAP is secure in the face of access privilege escalation as well as other known attacks. In addition, we perform formal security verification using the popular automated verification tool Proverif.

### 6.1. ROR Model-Based Formal Security Analysis

We consider random oracles under the formal security model, where the adversary/attacker A can make multiple oracle queries (this part was not considered in the previous conference).

(1) ROR model:

In the login and authentication phase of 3ECAP, three participants $U_{i}$, GW and $SD_{j}$ are involved in this process. The model considers the following.

Participants: The instances *i*, *k*, and *j* corresponding to the participants $U_{i}$, GW, and $SD_{j}$ can be denoted as $\omega_{U_{i}}^{i}$, $\omega_{GW}^{k}$, and $\omega_{SD_{j}}^{j}$, respectively, which are called oracles.

Accepted state: An instance $\omega^{i}$ is in the accept state, indicating that it has received the last message. Once the messages sent and received by $\omega^{i}$ are sequentially ordered, it forms the session identifier side of $\omega^{i}$ for the running session.

Partnering: Two instances, called $\omega^{i}$ and $\omega^{j}$, are partners if the following conditions are met: (1) $\omega^{i}$ and $\omega^{j}$ are in the accepted state; (2) $\omega^{i}$ and $\omega^{j}$ have the same session identification (sid), i.e., $sid_{\omega }^{i}=sid_{\omega }^{j}$; and (3) $\omega^{i}$’s partner identification (pid) is $\omega^{j}$ and vice versa.

Freshness: Two instances, called $\omega^{i}$ and $\omega^{j}$, are fresh if the key $sk_{ij}$ ($=sk_{ji}$) established between $U_{i}$ and $SD_{j}$ is not disclosed by adversary A through reveal query.

Adversary: Since the ROR model is based on the DY threat model, adversary A can fully control all messages transmitted in the network, which means that A can eavesdrop, modify, delete, forge, or inject messages between two entities.

Execute ($\omega^{i}$,$\omega^{k}$,$\omega^{j}$): The passive attack is modeled under this query, which allows A to intercept all communication records between participants $U_{i}$, GW, and $SD_{j}$.

Send ($\omega^{i}$,*m*): This query is considered an active attack, where A can send a message *m* to an instance $\omega^{i}$ and also receive a response message.

Reveal ($\omega^{i}$): When this query is executed, the session key $sk_{ij}$ (=$sk_{ji}$) established between $\omega^{i}$ and its partner is leaked to A.

CorruptSC ($\omega_{U_{i}}^{i}$): Once such a query is executed, the information stored in the smart card $SC_{i}$ of $U_{i}$ is disclosed to A.

CorruptSD ($\omega_{SD_{j}}^{j}$): Under this query, A can extract all the sensitive information stored in a sensor by a power analysis attack. Therefore, this query is modeled as an active attack. In addition, we also assume that both CorruptSC and CorruptSD provide a weak corruption model where the temporary keys and internal data of the instance are not corrupted.

Test ($\omega^{i}$): The semantic security of the session key sk (i.e., $sk_{ij}$ or $sk_{ji}$) established between instances can be modeled with this query. Once this query is executed, a coin *c* is tossed and the result is returned to A. If c=1, the instance returns sk or a random number of the same length as sk if c=0; otherwise, it returns a null value.

It is worth noting that, according to [34], we perform a limit on the number of queries for CorruptSC and CorruptSD queries. However, A is allowed to execute multiple Test queries. Furthermore, since GW is absolutely secure in the network, A cannot obtain any information from GW by Corrupt query. All participants and A have access to a one-way collision-resistant hash function h(·), which is modeled as a random oracle.

(2) Security Proof: The semantic security (or AKE security) of the session key SK in 3ECAP is given in Theorem 1. Furthermore, similar proofs [35] and [34] follow Theorem 1.

**Theorem** **1.** *If A is the adversary in polynomial time against 3ECAP in the RoR model, and $q_{h}$, $q_{s}$, and $q_{e}$ denote the number of Hash queries, Send queries, and Execute queries, respectively, then*

$$Adv_{3ECAP,D}^{\mathrm{AKE}}\leq \frac{q_{h}^{2}}{2^{l_{h}}}+\frac{{\left(q_{s}+q_{e}\right)}^{2}}{2^{l_{r}}}+2\max\left(C^{\prime }q_{s}^{s^{\prime }},\frac{q_{s}}{2^{l_{b}}},\lambda_{p}q_{s}\right)$$

where $l_{h}$, $l_{r}$, and $l_{b}$ refer to the length of the hash output, the length of the random number, and the length of the user bio-secret parameter $r_{i}$, respectively. D is denoted as the password space and obeys the Zipf distribution, and $C^{\prime }$ and $s^{\prime }$ are the parameters of Zipf.

**Proof.**  The security proof of the proposed protocol (3ECAP) is composed of a series of games: $G_{0}$, $G_{1}$, $G_{2}$, $G_{3}$. Suppose $Succ_{A}^{G_{j}}$ (j=0,1,2,3) represents an event in which A successfully guesses the random bit *c* of a tossed coin in the game $G_{j}$ and the corresponding probability of occurrence is denoted as $Pr[Succ_{j}]$. □

Game $G_{0}$: This is the initial game where A performs a real attack simulation on 3ECAP in the ROR model. Thus, according to the definition of semantic security, we have

(1) $$Adv_{3ECAP,D}^{\mathrm{AKE}}=\left|2Pr[Succ_{0}]-1\right|.$$

Game $G_{1}$: It corresponds to a passive attack implemented by A, where A can perform an Execute query and intercept all messages $M_{1}=\left\{A_{i},B_{i},C_{i},RSID_{j},T_{1}\right\}$, $M_{2}=\left\{D_{i},E_{i},F_{i},T_{2}\right\}$ and $M_{3}=\left\{G_{i},H_{i},J_{i},T_{3}\right\}$ transmitted in the public channel during the login and authentication phases of $U_{i}$. Once the game is over, A executes a Test query and discriminates the genuine sk from a random number based on the results returned by the query, where $sk=h(b_{i}∥d_{i}∥RSID_{j}∥c∥T_{3})$, $b_{i}=h(RID_{i}∥T_{1}∥a)$ and $d_{i}=h\left(k_{GWj}∥T_{2}\right)$. Therefore, A needs the secret information $RID_{i}$, $k_{GWj}$ and *a* to calculate the session key sk. However, this secret information cannot be obtained by A by eavesdropping on messages $M_{1}$, $M_{2}$ and $M_{3}$. Therefore, the probability of adversary A winning the game $G_{1}$ does not increase. Due to the indistinguishability of games $G_{0}$ and $G_{1}$, we have

(2) $$Pr\left[Succ_{1}\right]=Pr\left[Succ_{0}\right].$$

Game $G_{2}$: Game $G_{2}$ is modeled as an active attack where the primary goal of A is to attempt to convince participating nodes that the forged message is legitimate. Suppose that A performs $q_{h}$ number of Hash queries with the help of $q_{s}$ number of the Send queries. Based on the results of the birthday paradox, the collision probability of the Hash query is at most $\frac{q_{h}^{2}}{2^{l_{h}}}$. Since the random numbers *a*, *b* and *c* exist in messages $M_{1}$, $M_{2}$ and $M_{3}$, respectively, the collision probability of the random numbers is at most $\frac{{\left(q_{s}+q_{e}\right)}^{2}}{2^{l_{r}}}$. Hence, we obtain

(3) $$\left|Pr\left[Succ_{2}\right]-Pr\left[Succ_{1}\right]\right|\leq \frac{q_{h}^{2}}{2^{l_{h}}}+\frac{{\left(q_{s}+q_{e}\right)}^{2}}{2^{l_{r}}}.$$

Game $G_{3}$: This is the last game, where A executes CorruptSC and CorruptSD queries. Specifically, the information $\left\{P_{i},\beta_{i}^{*},SDL_{i}^{*},RID_{GW},RID_{i},p_{i}\right\}$ stored in $SC_{i}$ and the information $\left\{RSID_{j},k_{GWj}\right\}$ stored in $SD_{j}$ are obtained by A using CorruptSC and CorruptSD, respectively. Note that the pseudo-identity $RSID_{j}$ and $k_{GWj}$ of all sensors are different from each other. In 3ECAP, $U_{i}$ uses both password $PW_{i}$ and bio-information $BIO_{i}$ for authentication, which can be divided into two cases.

Case 1: Suppose that A attempts to guess the low entropy password using $q_{s}$ number of the send queries. Since the user’s password follows Zipf’s law [36,37], the probability of this case is $C^{\prime }q_{s}^{s^{\prime }}$.

Case 2: Assume that A tries to extract the biological key $r_{i}$ of $U_{i}$ from the obtained information. Since 3ECAP adopts the fuzzy extractor technique, A can only extract at most $l_{b}$ random bits, and the corresponding probability of guessing $r_{i}$ is approximately $2^{-l_{b}}$. In addition, we consider the probability of false positive $\lambda_{p}$ that occurs for biometric feature extraction. In general, for fingerprints, $\lambda_{p}\approx 2^{-14}$ [34].

Therefore, based on case 1 and case 2, it follows that

(4) $$\left|Pr\left[Succ_{3}\right]-Pr\left[Succ_{2}\right]\right|\leq \max\left(C^{\prime }q_{s}^{s^{\prime }},\frac{q_{s}}{2^{l_{b}}},\lambda_{p}q_{s}\right).$$

Since all queries are executed, A can only win the game by guessing bit *c*. This means that

(5) $$Pr\left[Succ_{3}\right]=\frac{1}{2}.$$

From (1) and (2), it is given that

(6) $$\frac{1}{2}Adv_{3ECAP,D}^{\mathrm{AKE}}=\left|Pr\left[Succ_{1}\right]-\frac{1}{2}\right|.$$

From (5) and (6), we have

(7) $$\frac{1}{2}Adv_{3ECAP,D}^{\mathrm{AKE}}=\left|Pr\left[Succ_{1}\right]-Pr\left[Succ_{3}\right]\right|.$$

Using the trigonometric inequality, we can obtain

(8) $$\begin{array}{c} \left|Pr\left[Succ_{1}\right]-Pr\left[Succ_{3}\right]\right|\leq \left|Pr\left[Succ_{1}\right]-Pr\left[Succ_{2}\right]\right|\\ +\left|Pr\left[Succ_{2}\right]-Pr\left[Succ_{3}\right]\right|. \end{array}$$

Finally, from (3), (4), (7) and (8), we have

$$Adv_{3ECAP,D}^{\mathrm{AKE}}\leq \frac{q_{h}^{2}}{2^{l_{h}}}+\frac{{\left(q_{s}+q_{e}\right)}^{2}}{2^{l_{r}}}+2\max\left(C^{\prime }q_{s}^{s^{\prime }},\frac{q_{s}}{2^{l_{b}}},\lambda_{p}q_{s}\right).$$

### 6.2. Informal Security Analysis

(1) Medical Staff Impersonation Attack: The adversary/attacker A who attempts to impersonate a legitimate medical staff needs to create a valid message $M_{1}=\left\{A_{i},B_{i},C_{i},RSID_{j},T_{1}\right\}$, where $A_{i}=RID_{i}⊕h\left(RID_{GW}∥T_{1}\right)$, $B_{i}=h(RID_{i}∥RID_{GW}∥T_{1})⊕b_{i}$, $C_{i}=h(b_{i}∥SDL_{i}∥RID_{GW}∥RSID_{j}∥T_{1})$. Even if A can generate the timestamp $T_{1}^{\prime }$ and the random number $a^{\prime }$, A cannot recover $M_{1}$ due to the lack of the key secret information $RID_{i}$, $RID_{GW}$, $b_{i}$ and $SDL_{i}^{\prime }$. This indicates that 3ECAP is secure against a user impersonation attack.

(2) Gateway Impersonation Attack: In order to become a legitimate node by impersonating GW, adversary A needs to create a message $M_{2}=\left\{D_{i},E_{i},F_{i},T_{2}\right\}$ to send to $SD_{j}$, where $D_{i}=b⊕h\left(k_{GWj}∥T_{2}\right)$, $E_{i}=b_{i}⊕h\left(b∥T_{2}\right)$, $F_{i}=h(RSID_{j}∥k_{GWj}∥b_{i}∥b∥T_{2})$. Even if A can generate the timestamp $T_{2}^{\prime }$ and the random number $b^{\prime }$, A will be unable to recover $M_{2}$ as the calculations of $\left\{D_{i},E_{i},F_{i}\right\}$ need the secret information $k_{GWj}$, $b_{i}$ and $RSID_{j}$. Thus, 3ECAP is protected in a gateway impersonation attack.

(3) Sensor Impersonation Attack: Suppose that A attempts to generate a message $M_{3}=\left\{G_{i},H_{i},J_{i},T_{3}\right\}$ on behalf of $SD_{j}$ to become a legitimate device node, where $G_{i}=c⊕b_{i}$, $H_{i}=c⊕d_{i}$, $J_{i}=h(RSID_{j}∥d_{i}∥c∥T_{3})$. Although A can generate timestamp $T_{3}^{\prime }$ and random number $c^{\prime }$ due to the absence of secret information $b_{i}$ and $d_{i}$, A also cannot recover $M_{3}$.

(4) Stolen Verifier Attack: Assume that A has stolen the medical staff’s smart card $SC_{i}$ and obtains the secret information $\left\{P_{i},\beta_{i}^{*},SDL_{i}^{*},RID_{GW},RID_{i},p_{i}\right\}$ stored in $SC_{i}$ using the power analysis attack, where $P_{i}=h\left(ID_{i}∥h\left(PW_{i}∥r_{i}\right)\right)$, $\beta_{i}^{*}=\beta_{i}⊕HP_{i}$, $SDL_{i}^{*}=SDL_{i}⊕h\left(HP_{i}∥\beta_{i}\right)$, $RID_{i}=h(ID_{i}∥ID_{GW}∥\beta_{i})$, $RID_{GW}=h\left(ID_{GW}∥x\right)$. Suppose A guesses a password $PW_{i}^{\prime }$ and attempts to verify its authenticity using known information. However, verifying $PW_{i}^{\prime }$ requires guessing both the identity $ID_{i}$ and the secret information $r_{i}$ of $U_{i}$, which is computationally difficult to achieve due to the collision-resistant property of h(·) (see Definition 1). Similarly, A cannot guess the bio-information $r_{i}$ correctly without $ID_{i}$ and $PW_{i}$. Moreover, it is not possible for A to compute other information, such as $\beta_{i}$ and $SDL_{i}$, in the absence of $HP_{i}$. Hence, 3ECAP is secure against a stolen smart card attack.

(5) Replay Attack: Suppose that adversary A intercepts messages $M_{1}$, $M_{2}$ and $M_{3}$ in a session and replays them after some time. The replay attack makes the participating nodes unable to recognize the authenticity of the messages and may lead to system breakdown as the number of replayed messages increases. However, due to the presence of timestamp *T* in $M_{1}$, $M_{2}$ and $M_{3}$, when a node receives a message, the first task for it is to verify the validity of *T* under the condition $\left|T^{*}-T\right|\leq \Delta T$, where $T^{*}$ represents the reception timestamp. Therefore, 3ECAP is secure against a replay attack.

(6) Denial-of-Service Attack: In the login and authentication phase of medical staff $U_{i}$, $U_{i}$ first inserts the smart card $SC_{i}$ and imprints his or her bio-information $BIO_{i}^{\prime }$ on the acquisition device, and also enters the corresponding identity $ID_{i}^{\prime }$ and password $PW_{i}^{\prime }$. If the condition $HamDis(BIO_{i}^{\prime },BIO_{i})\leq t$ is not satisfied, the whole session is terminated. Otherwise, $SC_{i}$ computes $r_{i}=Rep\left(BIO_{i}^{\prime },p_{i}\right)$, $P_{i}^{\prime }=h\left(ID_{i}^{\prime }∥h\left(PW_{i}^{\prime }∥r_{i}\right)\right)$, and verifies $P_{i}^{\prime }\overset{?}{=}P_{i}$. The session is also aborted if the equation does not hold. Therefore, it is clear that 3ECAP is capable of dealing with denial-of-service attacks.

(7) Sensor Device Capture Attack: Assume that A has captured $SD_{j}$ and obtained information $\left\{RSID_{j},k_{GWj}\right\}$ from it and attempts to compute the session key between $U_{i}$ and other uncaptured sensors $SD_{j}^{\prime }$ based on $\left\{RSID_{j},k_{GWj}\right\}$, where $RSID_{j}=h(SID_{j}∥ID_{GW}∥\alpha_{j})$, $k_{GWj}=h(ID_{GW}∥SID_{j}∥x)$. However, it is difficult for A to accomplish this task as these calculations require $SID_{j}$ and $\alpha_{j}$, which are randomly generated by GW. Hence, 3ECAP is secure in the face of a sensor device capture attack.

(8) Man-in-the-Middle Attack: In this attack, adversary A intercepts the messages $M_{1}$, $M_{2}$ and $M_{3}$ in a particular session and attempts to modify them into another form, which can make it impossible for participating nodes, such as $U_{i}$, GW, and $SD_{j}$, to determine whether they are communicating with a legitimate node. Suppose A intercepts message $M_{1}=\left\{A_{i},B_{i},C_{i},RSID_{j},T_{1}\right\}$ and forges a new message $M_{1}^{\prime }$ using the information in it, where $A_{i}=RID_{i}⊕h\left(RID_{GW}∥T_{1}\right)$, $B_{i}=h(RID_{i}∥RID_{GW}∥T_{1})⊕b_{i}$, $C_{i}=h(b_{i}∥SDL_{i}^{\prime }∥RID_{GW}∥RSID_{j}∥T_{1})$. Even if A has the ability to generate timestamp $T_{1}^{\prime }$ and random number $a^{\prime }$, A cannot forge message $M_{1}^{\prime }$, which can be recognized by participating nodes, due to the fact that these calculations require secret information $RID_{i}$, $RID_{GW}$, $b_{i}$ and $SDL_{i}^{\prime }$. Similarly, adversary A cannot forge $M_{2}^{\prime }$ and $M_{3}^{\prime }$. Therefore, 3ECAP is safe in responding to a man-in-the-middle attack.

(9) Insider Privilege Attack: There may be a scenario in which a privileged internal personnel of the trusted GW serves as an internal attacker A. This attack can be divided into two cases as follows.

Case 1: Assume that A obtains $RID_{i}$ of $U_{i}$ during the medical staff registration phase, where $RID_{i}=h(ID_{i}∥ID_{GW}∥\beta_{i})$. Without knowing the identity $ID_{i}$ of $U_{i}$ and the random number $\beta_{i}$, it is difficult for A to guess one of them correctly from $RID_{i}$ due to the collision resistance property of h(·).

Case 2: Suppose that A intercepts message $\left\{P_{i},RID_{i},SDL_{i},RID_{GW}\right\}$ at the time of medical staff registration, which is initially sent by GW to $U_{i}$ via a secure channel, where $P_{i}=h\left(ID_{i}∥RPW_{i}\right)$, $SDL_{i}=h(Ver_{i}∥T_{re}∥RID_{i})$, $RID_{GW}=h\left(ID_{GW}∥x\right)$. However, A cannot obtain any information from the message, due to the lack of $ID_{i}$, $RPW_{i}$, $Ver_{i}$, $T_{re}$, $ID_{GW}$, *x* and the collision resistance property of h(·). Hence, 3ECAP has the capability to cope with a privileged-insider attack.

(10) Privilege Escalation Attack: In this attack, medical staff $U_{i}$, authorized by GW, wants to gain data from other devices, which are out of $U_{i}$’s access list, by upgrading his/her access privilege. For this purpose, the access list AL for $U_{i}$ needs to be changed from $AL=\left\{RSID_{1},RSID_{2},...,RSID_{j}\right\}$ to $AL^{\prime }=\left\{RSID_{1}^{\prime },RSID_{2}^{\prime },...,RSID_{j}^{\prime }\right\}$, such that $AL^{\prime }\neq AL$ and $Ver_{i}^{\prime }=Ver_{i}$, when $U_{i}$’s sensor device list is $SDL_{i}=h(Ver_{i}∥T_{re}∥RID_{i})$. Although $U_{i}$ gains $T_{re}$, he or she cannot upgrade AL while keeping $SDL_{i}$ unchanged, as explained below.

Note: Let $f_{j}\left(\cdot \right)$ be a function for the calculation of the root hash of a merkle tree consisting of *j* leaf nodes. Also let $\left\{RSID_{1},RSID_{2},...,RSID_{j}\right\}$ be denoted as $\left\{D_{1},D_{2},...,D_{j}\right\}$. Then, we prove that $f_{j}\left(\cdot \right)$ has the property of collision resistance by mathematical induction, same as h(·). In order not to lose generality, assume that $j=2^{m}$ for m≥1. Given $AL=\left\{D_{1},D_{2}\right\}$ for m=1, we have

(9) $$f_{2}\left(D_{1},D_{2}\right)=h\left(H_{10}∥H_{11}\right)$$

where $H_{10}=D_{1}$ and $H_{11}=D_{2}$. It is obvious that $f_{2}\left(\cdot \right)$ is a collision-resistant function, same as h(·). Suppose the same is true when m=k, that is, there is no

(10) $$f_{2^{k}}\left(D_{1},D_{2},...,D_{2^{k}}\right)=f_{2^{k}}\left(D_{1}^{\prime },D_{2}^{\prime },...,D_{2^{k}}^{\prime }\right)$$

where $\left\{D_{1},D_{2},...,D_{j}\right\}\neq \left\{D_{1}^{\prime },D_{2}^{\prime },...,D_{j}^{\prime }\right\}$. Then, when m=k+1, we have

(11) $$f_{2^{k+1}}\left(D_{1},D_{2},...,D_{2^{k+1}}\right)=h\left(H_{10}∥H_{11}\right)$$

(12) $$H_{10}=f_{2^{k}}\left(D_{1},D_{2},...,D_{2^{k}}\right)$$

(13) $$H_{11}=f_{2^{k}}\left(D_{2^{k}+1},D_{2^{k}+2},...,D_{2^{k+1}}\right).$$

Therefore, it follows from (9), (10), (11), (12) and (13) that $f_{j}\left(\cdot \right)$ is as collision resistant as h(·). Let $AL=\left\{D_{1},D_{2},...,D_{j}\right\}$ and $AL^{\prime }=\left\{D_{1}^{\prime },D_{2}^{\prime },...,D_{j}^{\prime }\right\}$, where $j=2^{m}$ for m≥1 and $\left\{D_{1},D_{2},...,D_{j}\right\}\neq \left\{D_{1}^{\prime },D_{2}^{\prime },...,D_{j}^{\prime }\right\}$. Next, $Ver_{i}=f_{j}\left(D_{1},D_{2},...,D_{j}\right)$ and $Ver_{i}^{\prime }=f_{j}\left(D_{1}^{\prime },D_{2}^{\prime },...,D_{j}^{\prime }\right)$. Due to the collision-resistant nature of $f_{j}\left(\cdot \right)$, it is not feasible to find $AL^{\prime }$, where $AL^{\prime }\neq AL$, such that $Ver_{i}=Ver_{i}^{\prime }$ is satisfied.

Suppose $U_{i}$ obtains the registration timestamp $T_{re}$ and extracts the pseudo-identity $RID_{i}$ by power analysis attack, which is stored in $SC_{i}$. Then, $U_{i}$ expands the permissions to $AL^{\prime }=\left\{D_{1}^{\prime },D_{2}^{\prime },...,D_{j}^{\prime }\right\}$ and computes $Ver_{i}^{\prime }=f_{j}\left(D_{1}^{\prime },D_{2}^{\prime },...,D_{j}^{\prime }\right)$ and $SDL_{i}^{\prime }=h(Ver_{i}^{\prime }∥T_{re}∥RID_{i})$. However, in step 2.1 of the authentication phase, GW uses $SDL_{i}$ stored in the database to compute $C_{i}^{\prime }=h(b_{i}∥SDL_{i}∥RID_{GW}∥RSID_{j}∥T_{1})$ and verify $C_{i}^{\prime }\overset{?}{=}C_{i}$, where $C_{i}$ belongs to $M_{1}$ and is sent by $U_{i}$ to GW. It is clear that $C_{i}^{\prime }\neq C_{i}$ because of the collision-resistant property of $f_{j}\left(\cdot \right)$ and h(·) such that the whole session is terminated. Thus, 3ECAP is protected against a privilege escalation attack.

(11) Anonymity and Untraceability: In 3ECAP, all messages $M_{1}=\left\{A_{i},B_{i},C_{i},RSID_{j},T_{1}\right\}$, $M_{2}=\left\{D_{i},E_{i},F_{i},T_{2}\right\}$ and $M_{3}=\left\{G_{i},H_{i},J_{i},T_{3}\right\}$ of a particular session are set with timestamps $T_{1}$, $T_{2}$ and $T_{3}$, respectively, and also with random numbers *a*, *b* and *c*, which ensure that the participants $U_{i}$, GW and $SD_{j}$ in the session are not tracked by the adversary. Furthermore, 3ECAP uses pseudo-identities $RID_{i}$, $RID_{GW}$ and $RSID_{j}$ to transmit information in the public channel instead of the original identities $ID_{i}$, $ID_{GW}$ and $SID_{j}$, respectively, of the participating nodes in the session. Therefore, the anonymity of all participants in 3ECAP can be guaranteed.

### 6.3. Formal Verification with Proverif

Proverif is a formal automatic verification cryptographic protocol tool based on the Dolev–Yao model developed by Bruno Blanchet, which is able to describe various cryptographic primitives such as shared key cryptography and public key cryptography (encryption and digital signatures), Hash functions and Diffie–Hellman key exchange protocols. In addition, Proverif can handle an infinite session concurrent protocol and infinite message space, which overcomes the problem of state space explosion. When applying the Proverif tool to verify a cryptographic protocol, the tool gives a sequence of attacks if the protocol is vulnerable. All details about the usage of Proverif are in [38].

Four different channels, sch1, sch2, ch1 and ch2, are defined in Proverif, where sch1 and sch2 are secure channels for node registration and ch1 and ch2 are public channels for medical staff login and authentication. In addition, we define three processes for $U_{i}$, GW, and $SD_{j}$, respectively, and use process!User|!GW|!Device to implement the parallel operation of the three entities.

**Figure 5:**
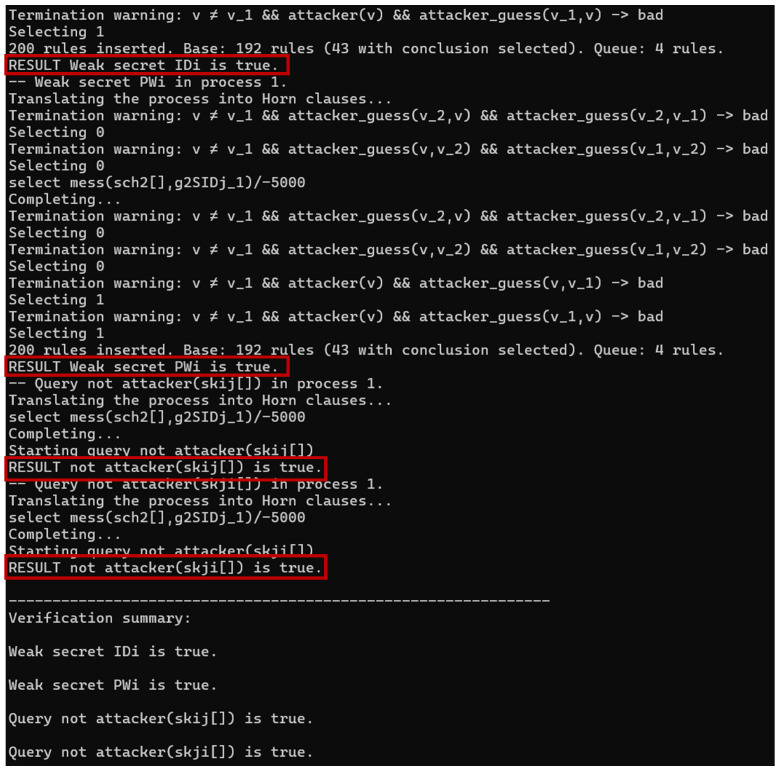
Results of executing Proverif.

The results of the Proverif execution are shown in Table 3 [30] and Figure 5. The first two rows demonstrate that both weak $ID_{i}$ and $PW_{i}$ can cope with guessing attacks. The last two rows imply that the generated session keys between $U_{i}$ and $SD_{j}$ are robust against common attacks. Therefore, 3ECAP is secure under formal verification.

## 7. Comparative Analysis

In this section, a comparative analysis of the calculation cost, communication and security features of 3ECAP and related protocols for Li et al. [26], Xie et al. [27], Son et al. [28] and Yang et al. [29] is shown.

### 7.1. Calculation Costs Comparison

**Figure 6:**
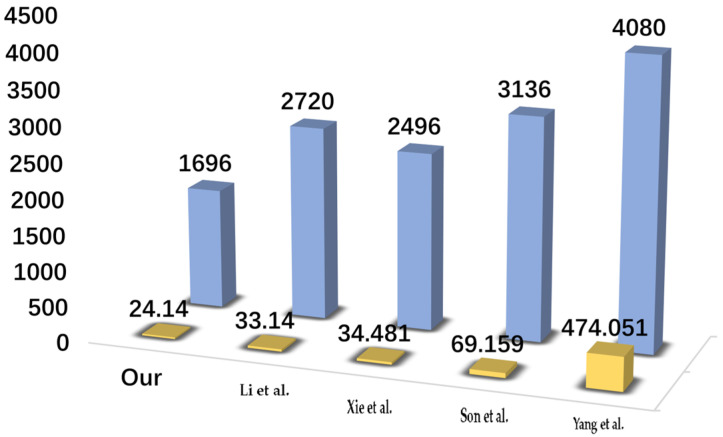
Comparison of calculation and communication cost [26,27,28,29].

The calculation costs required for 3ECAP and other protocols in the login and authentication phases are provided in this section. Assume that $T_{h}$, $T_{as}$, $T_{bp}$, $T_{ecc}$ and $T_{f}$ represent the time required for the hash function (SHA-256), asymmetric encryption/decryption (RSA-1024), bilinear pairing, ECC point multiplication and fuzzy extractor, respectively. Based on the available experimental results of Challa et al. [39], the time required to use these functions are $T_{h}=0.019$ ms, $T_{as}=19.536$ ms, $T_{bp}=44.517$ ms, $T_{ecc}=2.61$ ms and $T_{f}=1.71$ ms. Specifically, the various calculation costs required for the user, gateway, and sensor in each protocol are shown in Table 4 and Figure 6. The calculation cost of 3ECAP for the $U_{i}$, GW, and $SD_{j}$ are, respectively, $T_{f}+10T_{h}$, $6T_{h}$ and $6T_{h}$. The total calculation cost of 3ECAP is only 24.14 ms compared to other protocols, which is especially suitable for the communication requirements for IoMT.

### 7.2. Communication Costs Comparison

To measure the communication cost of the login and authentication phase, we assume that the identity, hash digest, random nonce, ECC point multiplication, asymmetric encryption/decryption (RSA-1024), and timestamp are 160 bits, 160 bits, 128 bits, 320 bits, 512 bits and 32 bits, respectively. Therefore, the total communication cost in 3ECAP is 1696 bits. The protocols of Li et al. [26], Xie et al. [27], Son et al. [28] and Yang et al. [29] require 2720, 2496, 3136, and 4080 bits (b) of communication cost, respectively. The details are shown in Table 5.

### 7.3. Security Features Comparison

The comparative analysis of thesecurity and functional features of 3ECAP and other related protocols is presented in Table 5. It can be observed that 3ECAP provides improved security and more functional features compared to the other four protocols. For example, the protocol by Li et al. [26] directly uses the identity of the participating nodes for information transmission, which can be easily tracked by the adversary. Moreover, in IoMT, the permission of different levels of users should be divided, which is not involved in the four other protocols. In contrast, 3ECAP divides several sensors into corresponding clusters based on the user’s access list and stores $SDL_{i}$ in a smart card and gateway, which not only achieves permission segmentation but also eliminates part of the subsequent database validation. Therefore, 3ECAP clearly outperforms other related protocols according to the comparison of all the features in Table 6.

## 8. NS3 Simulation

In this section, we attempt to measure the performance of 3ECAP in terms of network throughput (in bytes/second) and end-to-end delay (EED, in seconds) using the widely accepted NS3 tool (this part was not considered in the previous conference).

### 8.1. Simulation Parameters and Scenario

Table 7 [30] lists the basic network parameters used in the NS3 simulation. We used the Ubuntu 18.04.4 LTS platform. The simulation of the user, gateway, and sensor was executed on 2.4GHz Wi-Fi media. The gateway was set at the origin. The users were permitted to move randomly in any direction at a speed of 3 m within a 150 m^2^ area centered at the origin. Sensors were randomly distributed on an 80-m ring and centered on the gateway. We then set the size of the messages transmitted between the nodes, i.e., $M_{1}=84$ bytes, $M_{2}=64$ bytes, and $M_{3}=64$ bytes.

In this scenario, a complete message transfer consists of (the NS3 simulation does not involve specific cryptographic operations) (1) the user first sends an authentication request $M_{1}$ to a gateway in order to access the device; (2) the gateway receives the request and then forwards $M_{2}$ to the device; and (3) once it receives the message from gateway, the device sends the message $M_{3}$ to the user. Through 1, 2 and 3, the information interaction between a user and a device can be accomplished. Meanwhile, since there is more than one user and device in the scenario, they can all authenticate each other through the gateway. Therefore, it can be assumed that there are multiple message transfers at a given moment. And the main purpose of using NS3 is to show how the total throughput and delay change with the number of participating nodes.

**Figure 7:**
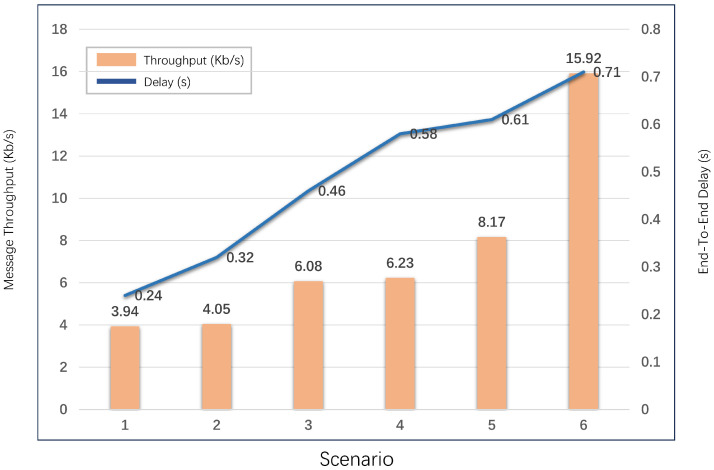
NS3-based 3ECAP simulation results.

We also set the simulation time for this scenario to 1200 s, which is a relatively appropriate setting that is sufficient to reflect the simulation results of 3ECAP. Finally, we configure a different number of users and devices, and the simulation parameters and results are shown in Table 7 [30] and Figure 7.

### 8.2. Discussion of Simulation Results

(1) Impact on Network Throughput: The total throughput of 3ECAP in the six scenarios is represented by bar charts in Figure 7. The throughput is calculated as $\varrho_{d}/\left(\sigma_{s}-\sigma_{r}\right)$, where the total amount of data received in the simulated environment is $\varrho_{d}$, the time to send the first packet is $\sigma_{s}$, and the time to receive the last packet is $\sigma_{r}$. It is observed that as the number of participating nodes, including users and sensors, increases, the network throughput in the network also increases accordingly.

(2) Impact on End-to-End Delay: The total delay of 3ECAP in the six scenarios is represented by the discounted graph in Figure 7. EED delay can be expressed as $\sum_{i=1}^{\nu_{p}}\left(T_{si}-T_{ri}\right)/\nu_{p}$, where $T_{si}$ and $T_{ri}$ represent the sending time and receiving time, respectively, when the *i*th packet is transmitted, and the total number of packets transmitted during the simulation is $\nu_{p}$. It follows from the figure that when the number of participating nodes increases, the number of messages transmitted will increase, which may cause network congestion to the extent that the EED delay increases.

## 9. Conclusions

Considering the aspects of security, low cost, and access control for IoMT sensors, in this paper, we propose a new efficient cluster-based user authentication protocol (3ECAP). In 3ECAP, three factors, i.e., password, biometric and smart card, are employed to resist a single-factor incidental guessing attack. In addition, 3ECAP enables user-specific privilege segmentation through fine-grained access control and can address the resulting privilege escalation attack. Furthermore, provable security based on the ROR model, formal verification based on the Proverif tool, as well as non-formal analysis are provided in this paper, and the results demonstrate the robustness of 3ECAP in the face of most attacks. Finally, the comparison and analysis with the latest related protocols indicate that 3ECAP provides higher security and lower computation and communication costs; therefore, it is very suitable for the practical deployment of the IoMT.

Future research directions related to this paper are as follows: (1) implementing and evaluating 3ECAP in real IoMT environments, (2) providing a flexible on-line sensor device addition phase, and (3) supporting dynamic updating of user-accessible lists based on sensor clusters in order to maintain forward and backward secrecy.

## Figures and Tables

**Table 1:** Related works.

Reference	Method	Advantage (+)	Limitation (−)
Wang et al. [13]	ECC, hash, fuzzy extractor	+three-factor authentication +forward secrecy	−high computational cost −user privacy −lack of access control
Masud et al. [14]	hash, password	+lightweight authentication +node anonymity	−impersonation attack −replay attack −lack of access control
Sutrala et al. [15]	ECC, hash	+impersonation attack protection +MITM attack protection	−privilege-insider attack −high computational cost −lack of access control
Iqbal et al. [16]	hash, symmetric encryption	+privacy-preserving +node anonymous	−impersonation attack −replay attack −lack of access control
Wei et al. [17]	ECC, hash, pseudo random function	+privacy-preserving +system secret key update	−impersonation attack −lack of access control
Zhang et al. [21]	hash, password, homomorphic encryption	+key leakage protection +anonymity and untraceability	−password guessing attack −lack of access control −high resource cost
Nandy et al. [22]	hash, ECC, RSA or DSA	+privacy-preserving +forward secrecy −insider attack protection	−clogging attack −high resource cost −lack of access control
Singh et al. [23]	hash, fuzzy extractor, PUF	+two-factor authentication +physical layer security	−MITM attack −replay attack −high resource cost −privilege escalation attack
Nyangaresi et al. [25]	hash, ECC, password	+replay attack protection +impersonation attack protection +MITM attack protection	−anonymity and untraceability −device capture attack −high resource cost −lack of access control
Li et al. [26]	hash, ECC	+three-factor authentication +forward secrecy +device capture attack protection +impersonation attack protection	−high resource cost −untraceability −lack of access control
Xie et al. [27]	hash, ECC, PUF	+device capture attack protection +MITM attack protection +impersonation attack protection	−privilege-insider attack −forward secrecy −lack of access control
Son et al. [28]	hash, ECC, password	+anonymity and untraceability +ephemeral key leakage protection	−device capture attack −high calculation cost −lack of access control
Yang et al. [29]	hash, ECC, bilinear pairing	+device update +token forgery attack protection	−device capture attack −privacy disclosure −high resource cost −lack of access control

**Table 2:** Notations and abbreviations.

Noation	Description
$U_{i}$, GW, $SD_{j}$	$i_{th}$ user, $j_{th}$ sensor and gateway
$ID_{i}$, $ID_{GW}$, $SID_{j}$	Identities of $U_{i}$, GW and $SD_{j}$
$RID_{i}$, $RID_{GW}$, $RSID_{j}$	Pseudo-identities of $U_{i}$, GW, and $SD_{j}$
$SC_{i}$, $BIO_{i}$	Smart card and biometrics of user
AL	User’s access list
Gen(·), Rep(·)	Functions of the fuzzy extractor
$r_{i}$, $p_{i}$	Secret parameter and public parameter of $U_{i}$
$HamDis(BIO_{i}^{\prime },BIO_{i})$	Hamming distance between $BIO_{i}^{\prime }$ and $BIO_{i}$
*t*	Fault tolerance threshold applied in Rep(·)
$\lambda_{n}$, $\lambda_{p}$	False negative probability and false positive probability
h(·)	One-way collision-resistant hash function
⊕, ∥	Bitwise XOR and concatenation operations
$T_{1}$, $T_{2}$, $T_{3}$	Current timestamps
ΔT	Maximum transmission delay
$\alpha_{j}$, $\beta_{i}$	Random numbers applied in the registration phase
*a*, *b*, *c*	Random numbers applied in the login and authentication phase
*x*	Master key for GW
$k_{GWj}$, $k_{jGW}$	Shared keys for GW and $SD_{j}$
A	Adversary
P→Q:M	*P* sends the message *M* to *Q*

**Table 3:** Results for code.

Secure channel	sch1, sch2
Public channel	ch1, ch2
Process	User, GW, Device
RESULT Weak secret IDi is true (bad not derivable).	
RESULT Weak secret PWi is true (bad not derivable).	
RESULT not attacker(skij[]) is true.	
RESULT not attacker(skji[]) is true.	

**Table 4:** Calculation costs comparison.

Protocol	User	Gateway	Sensor Device	Total Cost	Rough Estimation
3ECAP	$T_{f}+10T_{h}$	$6T_{h}$	$6T_{h}$	$T_{f}+22T_{h}$	24.14 ms
Li et al. [26]	$T_{f}+3T_{ecc}+8T_{h}$	$T_{ecc}+8T_{h}$	$2T_{ecc}+4T_{h}$	$T_{f}+6T_{ecc}+20T_{h}$	33.14 ms
Xie et al. [27]	$12T_{h}+5T_{ecc}$	$10T_{h}+6T_{ecc}$	$7T_{h}+2T_{ecc}$	$29T_{h}+13T_{ecc}$	34.481 ms
Son et al. [28]	$15T_{h}+3T_{ecc}$	$8T_{h}+3T_{ecc}+T_{as}$	$10T_{h}+2T_{as}$	$33T_{h}+6T_{ecc}+3T_{as}$	69.159 ms
Yang et al. [29]	$5T_{h}+7T_{ecc}+3T_{bp}$	$2T_{h}+2T_{ecc}+4T_{bp}$	$2T_{h}+2T_{ecc}+3T_{bp}$	$9T_{h}+11T_{ecc}+10T_{bp}$	474.051 ms

**Table 5:** Communication costs comparison.

Messages	3ECAP	[26]	[27]	[28]	[29]
$U_{i}\to GW$	672 b	800 b	960 b	672 b	684 b
$GW\to SD_{j}$	512 b	640 b	1088 b	672 b	2684 b
$SD_{j}\to GW$	—	640 b	—	—	—
$GW\to U_{i}$	—	640 b	—	—	—
$SD_{j}\to U_{i}$	512 b	—	448 b	1792 b	712 b
Total cost	1696 b	2720 b	2496 b	3136 b	4080 b

**Table 6:** Security features comparison.

Feature	3ECAP	[26]	[27]	[28]	[29]
User impersonation attack	✔	✔	✔	×	✔
Gateway impersonation attack	✔	✔	✔	×	✔
Sensor device impersonation attack	✔	✔	✔	×	✔
Stolen verifier attack	✔	—	✔	×	✔
Replay attack	✔	✔	✔	✔	✔
Denial-of-service attack	✔	✔	×	×	×
Sensor device capture attack	✔	×	✔	×	×
Man-in-the-middle attack	✔	×	✔	✔	✔
Insider privilege attack	✔	×	✔	×	✔
Privilege escalation attack	✔	×	×	×	×
Anonymity	✔	—	×	✔	×
Untraceability	✔	✔	×	×	×
Forward secrecy	✔	✔	✔	✔	×
Mutual authentication	✔	✔	✔	✔	×
Session key agreement	✔	×	✔	✔	✔
Biometric update	✔	✔	×	×	×
Password change	✔	✔	✔	×	×
Sensor device addition	✔	—	—	—	—
Two/three factor authentication	3	3	3	2	2
Fine-grained access control	✔	×	×	×	×
Formal analysis	✔	✔	✔	×	✔
Authentication based on Proverif/AVISPA tool	✔	✔	✔	×	×

✔: The protocol securely resists a particular attack or supports a particular feature; ×: the protocol is insecure against a particular attack or does not support a particular feature; —: not applied in the protocol.

**Table 7:** Simulation parameters.

Parameter	Description	
Platform	NS3 3.27/Ubuntu 18.04.4 LTS	
Mobility	random (3 m/s)	
Simulation time	1200 s	
Scenarios	No. of users	No. of devices
1	10	5
2	5	10
3	8	10
4	5	15
5	5	20
6	8	50

## Data Availability

Data are contained within the article.

## References

[1] Laghari A.A., Wu K., Laghari R.A., Ali M., Khan A.A. (2021). A review and state of art of Internet of Things (IoT). Arch. Comput. Methods Eng..

[2] Soori M., Arezoo B., Dastres R. (2023). Internet of things for smart factories in industry 4.0, a review. Internet Things Cyber-Phys. Syst..

[3] Sadhu P.K., Yanambaka V.P., Abdelgawad A. (2022). Internet of things: Security and solutions survey. Sensors.

[4] Zhang L., Lin Y., Yang X., Chen T., Cheng X., Cheng W. (2024). From Sample Poverty to Rich Feature Learning: A New Metric Learning Method for Few-Shot Classification. IEEE Access.

[5] Mahmoud H.H.H., Amer A.A., Ismail T. (2021). 6G: A comprehensive survey on technologies, applications, challenges, and research problems. Trans. Emerg. Telecommun. Technol..

[6] Tataria H., Shafi M., Molisch A.F., Dohler M., Sjöland H., Tufvesson F. (2021). 6G wireless systems: Vision, requirements, challenges, insights, and opportunities. Proc. IEEE.

[7] Razdan S., Sharma S. (2022). Internet of medical things (IoMT): Overview, emerging technologies, and case studies. IETE Tech. Rev..

[8] Hernandez-Jaimes M.L., Martinez-Cruz A., Ramírez-Gutiérrez K.A., Feregrino-Uribe C. (2023). Artificial intelligence for IoMT security: A review of intrusion detection systems, attacks, datasets and Cloud-Fog-Edge architectures. Internet Things.

[9] Garg N., Wazid M., Singh J., Singh D.P., Das A.K. (2022). Security in IoMT-driven smart healthcare: A comprehensive review and open challenges. Secur. Priv..

[10] Hireche R., Mansouri H., Pathan A.S.K. (2022). Security and privacy management in Internet of Medical Things (IoMT): A synthesis. J. Cybersecur. Priv..

[11] Koutras D., Stergiopoulos G., Dasaklis T., Kotzanikolaou P., Glynos D., Douligeris C. (2020). Security in IoMT communications: A survey. Sensors.

[12] Mishra N., Pandya S. (2021). Internet of things applications, security challenges, attacks, intrusion detection, and future visions: A systematic review. IEEE Access.

[13] Wang C., Wang D., Duan Y., Tao X. (2023). Secure and Lightweight User Authentication Scheme for Cloud-Assisted Internet of Things. IEEE Trans. Inf. Forensics Secur..

[14] Masud M., Gaba G.S., Choudhary K., Hossain M.S., Alhamid M.F., Muhammad G. (2022). Lightweight and Anonymity-Preserving User Authentication Scheme for IoT-Based Healthcare. IEEE Internet Things J..

[15] Sutrala A.K., Bagga P., Das A.K., Kumar N., Rodrigues J.J., Lorenz P. (2020). On the design of conditional privacy preserving batch verification-based authentication scheme for internet of vehicles deployment. IEEE Trans. Veh. Technol..

[16] Iqbal W., Abbas H., Deng P., Wan J., Rauf B., Abbas Y., Rashid I. (2020). ALAM: Anonymous lightweight authentication mechanism for SDN-enabled smart homes. IEEE Internet Things J..

[17] Wei L., Cui J., Xu Y., Cheng J., Zhong H. (2020). Secure and lightweight conditional privacy-preserving authentication for securing traffic emergency messages in VANETs. IEEE Trans. Inf. Forensics Secur..

[18] Yang Y., Huang X. (2021). Comments on “On the Design of Conditional Privacy Preserving Batch Verification-Based Authentication Scheme for Internet of Vehicles Deployment”. Cryptol. ePrint Arch..

[19] Yu S., Das A.K., Park Y. (2021). Comments on “ALAM: Anonymous lightweight authentication mechanism for SDN enabled smart homes”. IEEE Access.

[20] Zhang J., Zhang Q. (2021). Comment on “Secure and Lightweight Conditional Privacy-Preserving Authentication for Securing Traffic Emergency Messages in VANETs”. IEEE Trans. Inf. Forensics Secur..

[21] Zhang S., Liu Y., Gao T., Xie Y., Zhou C. (2024). Practical and Secure Password Authentication and Key Agreement Scheme Based Dual-Server for IoT Devices in 5G Network. IEEE Internet Things J..

[22] Nandy T., Idris M.Y.I., Noor R.M., Wahab A.W.A., Bhattacharyya S., Kolandaisamy R., Yahuza M. (2021). A Secure, Privacy-Preserving, and Lightweight Authentication Scheme for VANETs. IEEE Sens. J..

[23] Singh N., Das A.K. (2024). TFAS: Two factor authentication scheme for blockchain enabled IoMT using PUF and fuzzy extractor. J. Supercomput..

[24] Chaudhry S.A. (2022). Comments on “A Secure, Privacy-Preserving, and Lightweight Authentication Scheme for VANETs”. IEEE Sens. J..

[25] Nyangaresi V.O. ECC Based Authentication Scheme for Smart Homes. Proceedings of the 2021 International Symposium ELMAR.

[26] Li X., Peng J., Obaidat M.S., Wu F., Khan M.K., Chen C. (2019). A secure three-factor user authentication protocol with forward secrecy for wireless medical sensor network systems. IEEE Syst. J..

[27] Xie Q., Ding Z., Tang W., He D., Tan X. (2023). Provable Secure and Lightweight Blockchain-Based V2I Handover Authentication and V2V Broadcast Protocol for VANETs. IEEE Trans. Veh. Technol..

[28] Son S., Lee J., Park Y., Park Y., Das A.K. (2022). Design of Blockchain-Based Lightweight V2I Handover Authentication Protocol for VANET. IEEE Trans. Netw. Sci. Eng..

[29] Yang A., Weng J., Yang K., Huang C., Shen X. (2022). Delegating Authentication to Edge: A Decentralized Authentication Architecture for Vehicular Networks. IEEE Trans. Intell. Transp. Syst..

[30] Su X., Xu Y., Tong H., Li T. A Cluster-based User Authentication Protocol for Internet of Medical Things Deployment. Proceedings of the 2023 International Conference on Wireless Communications and Signal Processing (WCSP).

[31] Ebrahimi S., Bayat-Sarmadi S. (2021). Lightweight fuzzy extractor based on LPN for device and biometric authentication in IoT. IEEE Internet Things J..

[32] Dolev D., Yao A. (1983). On the security of public key protocols. IEEE Trans. Inf. Theory.

[33] Abdalla M., Fouque P.A., Pointcheval D. (2005). Password-based authenticated key exchange in the three-party setting. Proceedings of the International Workshop on Public Key Cryptography.

[34] Banerjee S., Odelu V., Das A.K., Srinivas J., Kumar N., Chattopadhyay S., Choo K.K.R. (2019). A provably secure and lightweight anonymous user authenticated session key exchange scheme for internet of things deployment. IEEE Internet Things J..

[35] Das A.K., Wazid M., Kumar N., Vasilakos A.V., Rodrigues J.J. (2018). Biometrics-based privacy-preserving user authentication scheme for cloud-based industrial Internet of Things deployment. IEEE Internet Things J..

[36] Roy S., Das A.K., Chatterjee S., Kumar N., Chattopadhyay S., Rodrigues J.J. (2018). Provably secure fine-grained data access control over multiple cloud servers in mobile cloud computing based healthcare applications. IEEE Trans. Ind. Inform..

[37] Wang D., Cheng H., Wang P., Huang X., Jian G. (2017). Zipf’s law in passwords. IEEE Trans. Inf. Forensics Secur..

[38] Blanchet B., Smyth B., Cheval V., Sylvestre M. (2018). ProVerif 2.00: Automatic cryptographic protocol verifier, user manual and tutorial. Version.

[39] Challa S., Wazid M., Das A.K., Kumar N., Reddy A.G., Yoon E.J., Yoo K.Y. (2017). Secure signature-based authenticated key establishment scheme for future IoT applications. IEEE Access.

